# Evaluation of a Web-Based Stress Management Application—a Feasibility Study

**DOI:** 10.1007/s41347-018-0044-8

**Published:** 2018-02-15

**Authors:** Caroline Eklund, Magnus L. Elfström, Yvonne Eriksson, Anne Söderlund

**Affiliations:** 1 School of Health, Care and Social Welfare, Mälardalen University, Box 883, 721 23 Västerås, Sweden; 2 School of Health, Care and Social Welfare, Mälardalen University, Eskilstuna, Sweden; 3 School of Innovation, Design and Engineering, Mälardalen University, Eskilstuna, Sweden

**Keywords:** Stress, Internet, Behavior change, Occupational stress, Health promotion, Feasibility

## Abstract

The aim of the current study was to investigate the feasibility of a Web-based program that promotes behavior change for stress-related problems in terms of the program’s acceptability, practicability, and any possible effects. In addition, the aim was also to study how appropriate and realistic the study’s process and resource management would be for conducting a randomized controlled trial. A convenience sample consisting of 14 individuals was recruited from a university in Sweden. The participants had access to the program for a duration of 9 weeks. Questionnaires were answered before accessing, during use of, and after completing the program. Both qualitative and quantitative data were collected and analyzed. The program was considered acceptable and practically feasible, though small adjustments have to be made. The program was considered time-consuming, extensive, and in need of some clarifications. Regarding process and resource management, the study participants required minimum support. It was difficult to identify the time point when to send out the process measures because the participants worked at their own pace. Also, one of the process measurements, the motivation to change, remained stable. With some adjustments to the instructions to the study participants and minor changes in the program, the intervention and study’s procedure were considered as feasible and can be carried out in a randomized controlled trial.

## Introduction

Recently, supporting the management of mental health problems through Web-based programs has become a more researched topic. Web-based self-management programs have shown to be effective in supporting individuals to change different health-related behaviors (del Pozo-Cruz et al. 2013; Söderlund et al. 2009; Williams et al. 2010). But adherence to these programs is often low. One systematic review showed that in average only 50% of the users complete such programs (Kelders et al. 2012). Tailoring the program to each user, high grade of interactivity, and building the intervention on a solid theoretical framework are considered key factors for effective Web-based interventions (Maricutoiu et al. 2014; Webb et al. 2010). However, in Web-based interventions for stress management, tailoring the program to each user is complex because of the variety of symptoms experienced. This type of tailoring requires different types of stress-management strategies (Ong et al. 2004). As a result, tailoring has seldom been used in stress-management programs.

To address the problems of the current self-management programs for stress management, a Web-based program called My Stress Control (MSC) was developed. The program was built using evidence from multiple fields, such as behavior change, stress management, and information design. MSC is a multi-tracked, tailored, and interactive platform that includes effective behavior change techniques and stress-management strategies. Information is delivered in several ways: texts, videos, and audio recordings. The interactivity is at a high level and includes assessments and assignments that contribute to the tailoring of and further advancement within the program.

When conducting a feasibility investigation, the important parameters for a planned, randomized controlled trial (RCT) can be identified, adjusted, and further developed to improve the chances of success in a larger, more costly study. Feasibility studies can help to identify what needs to be modified regarding the intervention as well as regarding the research methodology (Thabane et al. 2010). The reasons for conducting a feasibility study are numerous, including being able to analyze the study process, resource management such as the study participants’ need for support, and assess the study’s safety and effect on outcomes (Thabane et al. 2010). A set of criteria for evaluating Web-based behavior change interventions was proposed by West and Michie (2016). These criteria for evaluating Web-based behavior change interventions target the acceptability of the intervention to key stakeholders, the practicability of the intervention to be implemented at scale to the intended users, the effectiveness of the intervention, if the intervention could be implemented within a realistic budget, and safety regarding any side effects and equity aspects of the intervention. Thabane et al.’s (2010) reasons for conducting a feasibility study correspond to the criteria suggested by West and Michie (2016); however, the acceptability aspect is more prominent in the criteria proposed by West and Michie (2016), and the study’s methodological aspects are more prominent in the list of reasons suggested by Thabane et al. (2010).

The aim of the current study was to investigate the feasibility of a Web-based program that promotes behavior change for stress-related problems in terms of the program’s acceptability, practicability, and any possible effects. In addition, the aim was also to study how appropriate and realistic the study’s process and resource management would be for conducting a randomized controlled trial.

## Methods

This study had an explorative, prospective mixed method design.

### Participants

A convenience sample of 15 individuals with administration and teaching functions were recruited from a Swedish university. Inclusion criteria were the same as in the planned, randomized control trial: perceived stress score, measured with the Perceived Stress Scale (Cohen et al. 1983) of 17 or higher (Brinkborg et al. 2011), employed, 18–65 years old, able to speak and understand the Swedish language, and consented to take part in the study. Exclusion criteria were as follows: currently on sick leave or scoring 11 or more on either of the subscales on the Hospital Anxiety and Depression Scale (Zigmond and Snaith 1983). One participant was excluded from the study because this participant scored above the cutoff on the Hospital Anxiety and Depression Scale (HADS).

### Content of MSC

MSC is an information-rich, Web-based, tailored, and interactive stress-management program that was developed using the theories and evidence from multiple fields, such as stress management, behavior change, and informational design (Mollerup 2015). The program is fully automated and thus does not provide the user with any contact to a therapist. MSC is an extensive program built on a solid theoretical framework that consists of the Social Cognitive Theory (Bandura 1989), Theory of Reasoned Action and Theory of Planned Behavior (Madden et al. 1992), Transtheoretical Model and Stages of Change (Donovan et al. 1998; Evers et al. 2006), and the Transactional Theory of Stress and Coping (Folkman and Lazarus 1988). MSC contains interactive components and feedback in several forms; MSC is different from existing programs in the field because of its ability to tailor the content to each user with multi-tracked opportunities. MSC’s tailoring in the program is based on readiness to change for each stress-management strategy. It is also based on symptoms of stress the user is experiencing, thus guiding the user to different stress-management strategies. MSC consists of 12 modules: introduction, psychoeducation (with information on what stress is, symptoms of stress, how to lower stress, and prompts the user to make a functional behavioral analysis using an Antecedent-Behavior-Consequence model, or ABC model), ambivalence, five different modules for stress-management strategies (assertiveness training, relaxation, pleasant activity scheduling, time management, and cognitive restructuring), ambivalence for lifestyle changes, two lifestyle change modules (techniques and advice for better sleep and support for physical activity), and a maintenance module. Before logging into the program for the first time, the users are screened for stress levels with the PSS-14 (Cohen et al. 1983; Cohen and Williamson 1988) as well as for depression and anxiety with HADS (Bjelland et al. 2002; Zigmond and Snaith 1983). Behavior change techniques are integrated into every program module. In MSC, self-monitoring, goal setting, reevaluation of goals, feedback, and prompting intention to change formulation are the central techniques (Michie et al. 2009). These techniques (except intention to change formulation) are used in the program’s modules to help the user practice stress-management strategies. Reevaluation of goals is central in the last module on maintenance. Prompting intention to change formulation is central in the ambivalence modules. The information is provided in text, videos, and audio recordings. Tailoring in the program individualizes the self-management to the unique user based on individual assessment (Kreuter et al. 1999).

### Measures

#### E-mail Communication

Bugs identified by the users, problems related to their understanding of the platform’s function, and other comments were communicated to the research team via e-mail. The need for support and how to make support available were reviewed through the e-mails.

#### Acceptability Questionnaire

Concerning the acceptability of the program, a questionnaire for gathering the participants’ opinions and experiences of content, tailoring, feedback, their ability to complete the behavioral analysis, set goals, the program’s graphic and pedagogical design, and how the information was perceived was used. Measured with an 11-graded Likert scale where 0 means “did not at all” and 10 means “to a high degree,” the questionnaire contained 13 statements regarding how well MSC was perceived regarding the areas stated above. The program was regarded acceptable if the mean score for the questionnaire was 5 or higher. The questionnaire also had three “yes or no” response statements regarding the usability of the platform, as well as one question about time frame for going through the program and one for time taken to answer the questionnaires for pre, process, and post measures. Finally, there were four open-ended questions about possible improvements, what modules the participants took part in, and what the participants considered to be the “take-home messages” from the program.

The following reliable and valid questionnaires were used in the current study and will be used in the upcoming RCT:

- *Perceived Stress Scale (PSS)*: The PSS (Cohen et al. 1983) assesses the frequency of stress-related thoughts and feelings. The PSS has 14 items, with responses given on a 5-point scale ranging from “never” to “very often.” In seven of the items, the response “very often” indicates high stress, whereas in the other half of the measures, the items are reversed. Items are summed into a total score. The highest score in the PSS-14 is 56. High scores indicate a level high of stress.
- *The Coping Self-efficacy Scale (CSS)*: The CSS (Chesney et al. 2006) measures perceived self-efficacy for coping with stressors. The CSS contains 26 items about the beliefs of performing behaviors important to adaptive coping. The items on the CSS are scored on an 11-point scale where 0 means “cannot do at all” and 10 means “being certain that one can do it.” Items are summed into a total score. High scores indicate high self-efficacy to cope with stress when things are not going as planned. The highest attainable score is 260.
- *The Motivation for Change Questionnaire (MCQ)*: The MCQ (Grahn and Gard 2008) measures motivation for change in a life or work situation. The MCQ contains 48 items, forming seven subscales relating to life situations: social support in life, mastery, challenges, control, values, self-efficacy, and self-confidence. It also has six subscales that are related to the work situations: coworker support, supervisor support, challenges in work, job control, interactions in work, and job satisfaction. For each subscale, the median is calculated. High scores indicate a high level of motivation.
- *The short version of the QPS Nordic, the QPS Nordic 34+ (QPS)*: The QPS for psychological and social factors at work contains 37 items that are divided into 23 subscales and single questions. All the items are scored using a 5-point Likert scale (Dallner et al. 2000). The subscales measure the following areas: work demands, role expectations, control at work, predictability of work, mastery at work, social interactions, leadership, organizational culture, perception of teamwork, work satisfaction, and perception of stress. The mean for each subscale is calculated.
- *The shortened version of the Utrecht Work Engagement Scale (UWES)*: The UWES is a nine-item scale that measures a person’s engagement in his or her work. All items are scored using a 7-point Likert scale. The means for each subscale, as well as the mean for the total scale, are calculated. The UWES has three subscales (“vigor,” “dedication,” and “absorption”) and a total score ranging between 0 and 6 (Schaufeli et al. 2006). High scores indicate a high level of work engagement, which is negatively related to burnout (Montgomery et al. 2003).
- *The situational version of the Brief COPE Questionnaire*: Brief COPE (Carver et al. 1989) is a 28-item scale with a 4-point response scale for each item that ranges from “never” to “very often.” It measures 14 different coping strategies for handling stressful situations. In addition, two four-item emotional approach coping scales are embedded into Brief COPE (Stanton et al. 2000). The items for each subscale are summed, save for the two last subscales on coping through emotional processing and emotional expression, and for these two, the means of the items are calculated. For the first 14 subscales, the scores can range from 2 to 8. The scores for the last two subscales can range from 1 to 4.
- *Process and resource management*: Managing how and when to deliver the questionnaires during the intervention regarding process measures was investigated and discussed in the research group.

### Procedure

This feasibility study had the same procedure as the upcoming RCT will have, save for the randomization procedure, and the same validated and reliable questionnaires were used. One further difference from the planned RCT was that the participants were instructed to go through the program at a quick pace. They were also informed to work with different stress-management strategies parallel. These instructions were used because the study focused on feasibility issues related to MSC’s acceptability and practicability, not to achieve a behavior change, but also because the time for conducting the present study was limited. In the planned RCT, the instructions in the program will prompt the users to work with stress-management strategies and assignments for a certain number of days, for example, 1 week or 1 day depending on the assignment. The estimated time for going through the program in the RCT will be 2–4 months, depending on what parts of the program are recommended for the individual user.

Informed consent was obtained from all participants in the current study. After informed consent was received, the study participants received login information and the first round of questionnaires. The study participants had access to MSC for 9 weeks before the final questionnaires were distributed. The participants were prompted to try to finish the program before post measurements were sent out. Measurements were conducted before, during, and after the intervention. The CSS and PSS were used before, during, and after the trial period of MSC. The MCQ was used before and during the use of MSC. The questionnaire on the acceptability of the program was used after the program. All other questionnaires were used before and after. Reminders to send in questionnaires were distributed after 1 week of each round of questionnaires. During the study period, the participants were encouraged to send comments and questions about bugs and usability problems to the first author. Some also contacted the first author in person and provided their verbal comments.

### Data Analyses

Descriptive statistics were used for demographic data as well as the questionnaire regarding the acceptability items. Inferential statistics were used to describe the possible pre- and post-MSC program changes. Bonferroni correction was used in the effect analyses. The open answers on the feasibility questionnaire regarding the program’s acceptability and practicability, e-mail conversations, and any verbal comments were categorized. Because the comments were short, often written in bullet-point format, they were not condensed into meaning units.

In total, on different questionnaires, there were four missing items from three different people. These items were replaced using the mean for the individual’s scoring on the scale or subscale. Two of the process questionnaires were not filled in at all (PSS and CSS) by one participant.

## Results

Fourteen participants completed the first round of measures. Ten participants completed the second round. Eight participants completed the third round. See Table 1 for the demographic data of the participants.

**Table 1:** Demographic data

Demographics (*n* = 14)	Medians and frequencies
Age (years)	44.5 (29–63)
Marital status	
Living alone	4 (29%)
Living with a partner	2 (14%)
Married	8 (57%)
Gender (% female)	13 (93%)
Education	
High school	2 (14%)
Bachelor/exam from university	7 (50%)
Masters	4 (29%)
Doctor	1 (7%)
Children living at home (median)	1 (0–2)
Age of children living at home	4.25 years (1–11)
% Work of full time (median)	
100%	9 (64%)
85%	2 (14%)
80%	1 (7%)
75	1 (7%)
50%	1 (7%)
Employment status	
Full-time employment	12 (86%)
Temporary employment	2 (14%)
Labor sector	State (100%)
Employed also by another employer than current	0%
Supervisory position (% yes)	2 (14%)

The main reason for dropping out of the study was a perceived shortage of time (four people). One study participant ended the study participation because of sick leave caused by depression and anxiety. One person did not send in the third round of measures and did not provide any reason for this.

### Acceptability and Practicability

Concerning the acceptability of the program, the results for the statements assessing the participants’ opinions and experiences of the content, tailoring, feedback, to conduct an own behavioral analysis, setting goals, graphic and pedagogical design, and how the information was perceived are summarized in Table 2. The median values for the eight participants were for most items over 5, which was the cutoff score for defining whether MSC was acceptable and practical. Understanding of the information regarding how to rehearse stress-management techniques and to understand how to formulate an ABC model (behavioral analysis) received the highest scores; both had a score of 8 on the 11-point Likert scale. The lowest score—a 4—came from the question on how the ambivalence module motivated users to continue to work with a stress-management strategy. Low scores were also given to how the “pop-up feedback” motivated the users to continue with the program, receiving a 4.5 on the 11-point Likert scale.

**Table 2:** Questionnaire results regarding acceptability and practicability of MSC reported for each study participant and the medians for each statement. The answers range from 0 (“not at all”) to 10 (“very much”)

Participants	It was easy to navigate the program	The program was tailored according to my perceived stress-related problems	The symptom of stress survey caught what I perceive related to my stress	Clarity regarding how the content was divided in a general introduction, tailored stress management strategies, lifestyle changes, and maintenance modules	It was possible to formulate my own ABC model with support by the program	I returned to my ABC model and used the situation described in the model when rehearsing my stress-management strategies	The information in text, pictures, and audio was easy to understand and made it easy to rehearse stress management	I felt that I could identify with the examples, pictures, and texts included in the program	I understood how I could use the material connected to the stress-management strategies	I was able to set goals that I could achieve with the support of the program	The last part of the program about seeking social support for stress management, recipe for future stress-management, set new goals, and make a summary prepared me for coping with stress in the future	I perceived that the pop-up feedback included in the program made it more likely for me to continue the program	If you went through ambivalence module: the module contributed to a decision to start the stress-management technique	Could you log in to MSC without contacting the support?	I was allowed access after PSS and HADS	I skipped recommended parts
1	8	7	4	6	10	8	10	6	9	9	6	0		Yes	Yes	Yes
2	7	8	8	7	8	6	8	8	8	7	7	7	6	No	Yes	No
3	10	10	10	9	8	8	8	8	8	9	9	7		Yes	Yes	No
4	3	3	3	8	3	2	8	3	7	2	3	2	2	Yes	Yes	Yes
5	8	6	7	9	10	5	9	7	8	5	6	7		Yes	Yes	No
6	7	9	7	9	8	0	6	3	4	3	5	4		Yes	Yes	No
7	9	9	9	5	8	5	10	8	5	5	5	5		Yes	Yes	Yes
8	7	6	5	7	6	1	7	7	6	3	3	2		Yes	Yes	Yes
Median	7.5	7.5	7	7.5	8	5	8	7	7.5	5	5.5	4.5	4	Yes	Yes	50/50

Six participants answered the question regarding the time spent for going through the program. The reported time ranged from 1 h to 7 weeks. The total amount of time spent on questionnaires for the pre, process, and post measures were answered by seven persons. The average time spent were 80 min, ranging from 60 to 120 min.

Regarding the open-ended answers on the acceptability questionnaire and the e-mail correspondence, six categories were identified regarding acceptability and practicability: extensiveness, interference, clarity, flexibility, insights, and need for reminders.

#### Extensiveness

The participants expressed that the program was more extensive than they first thought and that there was “too much” material to be filled in for the assignments on the stress-management strategies. It was suggested to make the assignments shorter. The program was perceived as time-consuming. One study participant described it as paradoxical: it is hard to take time for stress management when you already are stressed.

#### Interference

Some factors that interfered with using the platform were identified. One bug that locked further progress was found. Also, two of the audio recordings had low quality, and some of the videos lagged for one user. The video platform YouTube was used for embedding videos on MSC. However, it was perceived as confusing the way in which YouTube suggested other videos after the embedded video played. It was not clear for the users if these videos were included in the program or not.

#### Clarity

The need for clarifying some parts were identified. Here, the comments ranged from clarification of how items on the questionnaires were formulated to how the platform was constructed. The comments on the structure of the platform mainly concerned how easy or difficult it was to find a way through the program and how the program and platform signaled what was already completed and what was next. The small notifications when different sections of the MSC were completed were appreciated. The voices and pictures in the videos were perceived as suitable. Two study participants commented on the voices, which they liked. One voice was described as “kind, trustworthy, and supportive.” Suggestions on how to clarify progression through MSC by including instructions for navigation were provided. There was also conflicting information: in one part, MSC stated after a video to click on the “first activity,” and then beside the same activity, MSC stated “next activity.” Thus, more consistent use of commands was proposed to enhance clarity. One user wanted to look around at the platform before starting and was confused when it was impossible to reach the locked areas that would be unlocked later on. After watching the first video, the button “I have seen this film” was hidden by the scroll bar function, and the user did not understand that this button must be clicked to access the next task. One study participant noticed and liked the pop-up feedback saying that recommended stress-management strategies were available.

#### Flexibility

To enhance flexibility, participants suggested MSC should be delivered as a mobile application. MSC’s structure forces users to go through all the assignments in a predetermined order, and this was perceived as a barrier for using. One of the participants expressed that the individualization was not relevant. One of the participants with low stress levels expressed that for a person with a low stress level, it was difficult to stay motivated to go through such an extensive program.

#### Insights

The study participants described the insights they discovered regarding what stress really is for them and how their stress could be handled. Mainly, the insights were about becoming aware of their stress and how stress can be different for everyone. How to prevent stress and plan for recovery were also insights that the study participants shared.

#### Need for Reminders

The study participants expressed that they often forgot to log in to the program, and one participant suggested to add in reminders that could pop up on the screen.

### Possible Effects of the Program

The descriptive statistics (medians and range) for pre, process, and post measures for all the participants in all measures are presented in Table 3. The PSS-14 shows the points decreased from pre, to process, and to post measures. Likewise, the CSS shows points to be increasing during the same time. Most of the other variables seemed stable.

**Table 3:** Medians and range for pre, process, and post measures

Variables (min–max)	Median for pre measures (*n* = 14)	IRQ for pre measures (25th percentile: 75th percentile)	Median for process measures (*n* = 10)	IRQ for process measures (25th percentile: 75th percentile)	Median for post measures (*n* = 8)	IRQ for post measures (25th percentile: 75th percentile)
Perceived Stress Scale-14 (0–56)	26	22: 28	21	17.5: 29.5	20.5	16.5: 24.5
Coping Self-Efficacy Scale (0–260)	122	118: 141	135	118.5: 182.5	166	125.5: 191.5
Motivation to Change Questionnaire (1–4 for each subscale)						
Social support in life	3	3: 3	3	3: 3.5	NA	NA
Control in life	3	3: 3	3	3: 3	NA	NA
Competence to cope	3	3: 3	3	3: 3	NA	NA
Challenges in life	3	3: 3	3	3: 3.5	NA	NA
Goals/values	3	3: 3	3	3: 3	NA	NA
Self-efficacy	3	3: 4	3	3: 4	NA	NA
Self-confidence	3.5	3: 4	3.5	3: 4	NA	NA
Support from colleagues	3	3: 3	3	3: 3	NA	NA
Support from employer	3	3: 3	3	3: 3	NA	NA
Challenges at work	3	2.5: 3.5	3	3: 3	NA	NA
Control at work	3	3: 3	3	3: 3	NA	NA
Interaction	3	3: 3	3	3: 3	NA	NA
Goals	3.25	3: 3.5	3	3: 3.5	NA	NA
Utrecht Work Engagement Scale (0–6 for total and each subscale)	4.11	3.44: 4.56	NA	NA	4.11	3.78: 4.78
Vigor	4	3.33: 4.67	NA	NA	4.17	3.67: 4.84
Dedication	4.5	3.67: 4.67	NA	NA	4.33	4: 5.17
Absorption	4.33	3: 4.67	NA	NA	3.67	3.17: 5
Brief COPE Questionnaire (see each subscale for min and max)						
Self-distraction (2–8)	5	4: 6	NA	NA	5	4.5: 6.5
Active coping (2–8)	6	5: 6	NA	NA	6	6: 6.5
Denial (2–8)	2	2.5: 4	NA	NA	3	2: 4.5
Substance use (2–8)	2	2: 2	NA	NA	2	2: 2
Use of emotional support (2–8)	6	4: 6	NA	NA	6	6: 6.5
Use of instrumental support (2–8)	5	4: 6	NA	NA	6	5: 6
Behavioral disengagement (2–8)	2	2: 3	NA	NA	3	2: 4
Venting (2–8)	5	4: 7	NA	NA	6	5.5: 7
Positive reframing (2–8)	6	4: 6	NA	NA	6	5: 6
Planning (2–8)	6	4: 7	NA	NA	6	6: 7
Humor (2–8)	4	3: 6	NA	NA	4	4: 5
Acceptance (2–8)	6	5: 7	NA	NA	6	5.5: 6.5
Religion (2–8)	2	2: 2	NA	NA	2	2: 3
Self-blame (2–8)	4.5	3: 6	NA	NA	4	3.5: 4.5
Coping through emotional processing (1–4)	2.63	2: 3	NA	NA	2.75	2.63: 2.88
Coping through emotional expression (1–4)	2.63	2: 3	NA	NA	2.75	2.5: 3.25
QPS-Nordic 34+ (1–5 for each subscale)						
Quantitative demands	3.5	3: 4.5	NA	NA	3.25	3: 4.25
Demands on learning	2.25	2: 3	NA	NA	1.75	1.5: 2.5
Role clarity	4	3.5: 4.5	NA	NA	4	3.75: 4.75
Role conflicts	2	2: 2	NA	NA	2.5	2: 3
Positive challenges at work	4.5	3.5: 4.5	NA	NA	4.5	4: 4.5
Control over decisions	3	2.5: 3.5	NA	NA	3	2.5: 3.5
Control over working pace	4	3.4: 4.5	NA	NA	4	3.25: 4.25
Predictability over next month	4	4: 4	NA	NA	4	4: 4.5
Predictability (single item)	3	3: 4	NA	NA	3	3: 4
Experience of mastery	4	4: 4	NA	NA	4	4:4
Support from employer	3.75	3: 4.5	NA	NA	4	3: 4.5
Support from colleagues	4	4: 5	NA	NA	4	3.5: 4.5
Support from friends and family	4	4: 5	NA	NA	4	3.5: 4.5
Social interaction (single item)	3	3: 4	NA	NA	3	2: 3.5
Encouraging leadership	3	2.5: 3.5	NA	NA	3.25	2.75: 3.5
Social climate	4	2.5: 4.5	NA	NA	4	4: 4.75
Innovative climate	3.5	3: 4.5	NA	NA	4	3.75: 4.5
Inequality	2	1: 4.5	NA	NA	2	1: 2.75
Personnel targets	3	2.5: 4	NA	NA	3	2.5: 3.25
Organizational culture and climate (single item)	3	2: 4	NA	NA	4	3: 4
Teamwork	4.5	3.5: 4.25	NA	NA	4.5	3.75: 4.75
Work satisfaction	3.25	3: 3.5	NA	NA	3.5	3: 4.25
Stress (single item)	3	3: 4	NA	NA	3	2.5: 4.5

There were no significant differences between pre and post measures, save for one variable with *p* < 0.05: the coping strategy “acceptance.” However, when correcting for multiple tests according to Bonferroni, a significant *p* value for the variable “acceptance” should have been < 0.003. See Table 4 for detailed results from the inferential analyses.

**Table 4:** Differences between pre and post measures (*n* = 8)

Variables	z value	*p* value
Perceived Stress Scale-14	− 1.26	0.21
Coping Self-Efficacy Scale	− 1.40	0.16
Utrecht Work Engagement Scale total	− 0.35	0.73
Vigor	− 0.34	0.73
Dedication	− 0.17	0.87
Absorption	− 0.35	0.72
Brief COPE Questionnaire		
Self-distraction	− 0.96	0.34
Active coping	− 1.73	0.08
Denial	0.00	1.00
Substance use	0.00	1.00
Use of emotional support	− 1.73	0.08
Use of instrumental support	− 1.19	0.24
Behavioral disengagement	− 1.73	0.08
Venting	− 1.08	0.28
Positive reframing	− 0.73	0.79
Planning	− 1.75	0.08
Humor	− 1.22	0.22
Acceptance	− 2.12	0.03
Religion	− 1.00	0.32
Self-blame	0.00	1.00
Coping through emotional processing	− 0.32	0.75
Coping through emotional expression	− 0.54	0.59
QPS-Nordic 34+		
Quantitative demands	− 1.34	0.18
Demands on learning	− 1.51	0.13
Role clarity	− 0.45	0.66
Role conflicts	− 1.13	0.26
Positive challenges at work	0.00	1.00
Control over decisions	− 0.62	0.53
Control over working pace	0.00	1.00
Predictability over next month	− 0.58	0.56
Predictability (single item)	− 0.58	0.56
Experience of mastery	0.00	1.00
Support from employer	− 0.28	0.78
Support from colleagues	− 0.38	0.71
Support from friends and family	− 1.63	0.10
Social interaction (single item)	− 1.13	0.26
Encouraging leadership	− 0.74	0.46
Social climate	− 1.29	0.20
Innovative climate	− 0.88	0.38
Inequality	− 0.73	0.46
Personnel targets	− 0.43	0.67
Organizational culture and climate (single item)	− 1.89	0.06
Teamwork	− 1.00	0.32
Work satisfaction	− 1.41	0.16
Stress (single item)	− 0.71	0.48

### Process and Resource Management

There were some issues in determining the appropriate time to send out the process measures because the users worked at their own pace. Because of this, an overview function in the administration tool for MSC was developed during the current study, allowing the first author to see when the users had completed the recommended stress-management strategies and started the lifestyle-related strategies for stress management (advice to improve sleep and physical activity).

The study participants required minimum support while using the program.

Of the three process measures (the PSS, MCQ, and SES) included in the study, the MCQ was stable according to the median values (see Table 3).

## Discussion

MSC was found to be feasible when it came to the program’s acceptability and practicability. The study process and resource management were also considered to be feasible. However, the comments from the participants revealed how the program can be further developed for enhancing the chance for success in a RCT. Criteria for assessing acceptability were set before the study was conducted. This can be considered a strength. When criteria are set beforehand, the results may be less biased. The criteria for acceptability were set to relatively low cutoff scores because this was a pioneer study and explorative in nature. Most parameters had a mean well above the set cutoff score; thus, the criteria could have been higher. Two of the statements were below the cutoff score (scoring a 4 and a 4.5). The reason behind these low scores on MSC’s ambivalence module could be because only two participants took the ambivalence module and hence were the only ones who answered this question. After analyzing the questionnaire regarding MSC’s practicability and acceptability, there was a difference regarding the scores for two types of items: the questions related to issues that could be easily recognized earlier on, such as how easy the program was to navigate, how the program was individually tailored, and how the symptoms of stress survey caught the user’s stress-related symptoms, received higher scores. Questions regarding issues that would have required the user to work with the program longer, such as fulfilling goals and trying a new behavior in different situations, received lower scores. This could be because of the users’ shorter time with the program compared to what MSC was developed for and maybe because users were not expected to have enough time to fulfill their goals, hence not having time to try their new behavior in the situations described in their ABC model. Also, regarding the program’s acceptability, one participant scored above the cutoff score on HADS and excluded. However, the text with further instructions to contact other healthcare providers after being denied access was judged as fair and “okay” by that user.

The time spent going through the program varied a lot. The data provided by the study participants were difficult to decipher since it seems that the participants’ interpretation of the question was not clear. While one person may have answered how long time was spent on the Web-based platform, others seem to have answered how long time it took from first login, going through assignments provided by MSC until reaching the last module. How the question was formulated is probably a limitation in this study.

The current feasibility study was theoretically based on a model for developing and evaluating Web-based behavior change interventions (West and Michie 2016). Assessing the acceptability and practicability of a Web-based intervention on a smaller scale is preferable because doing so can help identify issues that can be fixed to enhance success in a larger, costlier RCT. Thus, in the RCT, the effect of a previously tested program would be the only focus because issues related to the program’s acceptability and practicability have already been investigated and corrected.

Behavior change takes time and does not occur overnight. The perceived extensiveness of the program was pointed out by several users. Even if difficulties with an extensive program such as MSC for people already experiencing high levels of stress have been shown, caution must be taken before making major changes in the program. The program, built on a solid theoretical frame and using evidence-based stress-management strategies and behavior change techniques, can not easily be condensed into a “shorter” program without losing its core ability to affect behavior change. One way to handle this issue could be to optimize the information in the beginning of the program and clarify the rationale and presumed effects of the program, as well as the quid pro quo demanded form the users. This could be done by enhancing and developing both the informational design in the earlier parts of the program and the psychoeducation section (Van Daele et al. 2012). The contradiction of being stressed about a lack of time and having to set aside time for stress management can be seen as an illustration of dysfunctional coping. Time-management problems, negative thinking and thinking errors, and not thinking of one’s own needs as important as another person’s needs could be reasons for not prioritizing one’s stress-management training. One limitation of the current study was the time limit. The participants were ambitious and worked with the program at a similar pace to what was planned for the RCT, which was not expected for this feasibility study. The information letter about the current study should have more clearly described the expectations of the study participants and how to use the program.

Analysis of the comments and feedback provided by the users indicated that several participants might not have completed the full program. This will be further investigated in an interview study regarding the users’ experiences of going through the program.

There is no definitive conclusion regarding the sample size in feasibility and pilot studies (Hertzog 2008; Julious 2005; Lancaster et al. 2002; Thabane et al. 2010). Judging the sample size must be based on the parameters to be estimated and the research focus. Studies have indicated population sizes from 10 individuals as being sufficient for assessing the clarity of instructions (Hertzog 2008); calculations for the precision of the mean and variance were found to require 12 (Julious 2005), and other studies have set the size to 30 or more (Lancaster et al. 2002). In the present study, the sample size was set to 15 individuals because that number seemed to be sufficient to estimate the feasibility issues (Julious 2005). One study on virtual reality-based stress-management training presented a significant effect (e.g., *p* < 0.001) even though the study population consisted of only a few people (*n* = 22) (Shah et al. 2015). This was not the case in the current study. Although, the medians of two of the outcome measures changed for the better after the program (see Table 3), the results of the current study did not indicate that MSC had a significant effect on any of the scales. The sample size was too small to draw any conclusions on the program’s possible effects. Also, the limited time for the study participants could have played a role in the absence of any effect. However, the main purpose of the present study was to investigate the feasibility issues, not the treatment effect.

Dropout rates were similar in the current study as in previous studies on Web-based behavior change interventions (Kelders et al. 2012). The study participants dropped out of the study mainly because of time limitations. The time limit to go through the program, based on the shortest time expected for going through the program in the planned RCT, might have caused some participants to perceive MSC as time-consuming, extensive, and inflexible, thus increasing the dropout rate. The fact that the program was perceived as extensive and time-consuming could also be linked to flexibility issues. For example, regarding flexibility, if the program could be adapted to the interface of a smartphone, the audio recordings, videos, and other materials could be viewed even if the user does not have access to a computer or tablet. The program could also be perceived as less extensive and more flexible if the users had more clearly understood that they could use modules, assignments, and connected materials that they thought were interesting, not only materials recommended by the program that emanated from their individually described symptoms. Nevertheless, the participants who dropped out during the study provided important feedback that contributed to the results of the present study.

Regarding the process and resource management (Thabane et al. 2010), there were some difficulties determining adequate timing when to send out the process measures because the study participants worked at their own pace. Therefore, an administration tool was developed. The resources concerning availability for support were estimated as feasible, and the participants required limited support to handle the program. The bug identified by one user and rectified by the programmer is not expected to reoccur. The MCQ was decided irrelevant for process measures in the upcoming RCT because it was stable in the pre and process measures. On the other hand, because there were only 10 participants conducting the process measures, this conclusion cannot be drawn for certain. This decision will be eligible for further discussions in the planning of the upcoming RCT. All the measures used were reliable and valid; they were also widely used, thus enhancing the ability to compare the results of the current study to others in the field.

The participants in this study were largely female (93%) and had a college education. This is considered as limitation regarding generalizability of the acceptability and feasibility to different populations. This must be considered when recruiting for the RCT.

## Conclusion

According to the predetermined cutoff scores, the current study shows that MSC is feasible for a RCT. However, to enhance the chance for success, some modifications need to be made before conducting the RCT.
